# Keratin–Chitosan Microcapsules via Membrane Emulsification and Interfacial Complexation

**DOI:** 10.1021/acssuschemeng.1c05304

**Published:** 2021-12-01

**Authors:** Amy Wilson, Ekanem E. Ekanem, Davide Mattia, Karen J. Edler, Janet L. Scott

**Affiliations:** † Department of Chemistry, University of Bath, Claverton Down, Bath BA2 7AY, United Kingdom; ‡ Department of Chemical Engineering and Centre for Advanced Separations Engineering, University of Bath, Claverton Down, Bath BA2 7AY, United Kingdom

**Keywords:** microencapsulation, membrane emulsification, keratin, chitosan, biopolymer, coacervation, layer-by-layer, polyelectrolyte complex

## Abstract

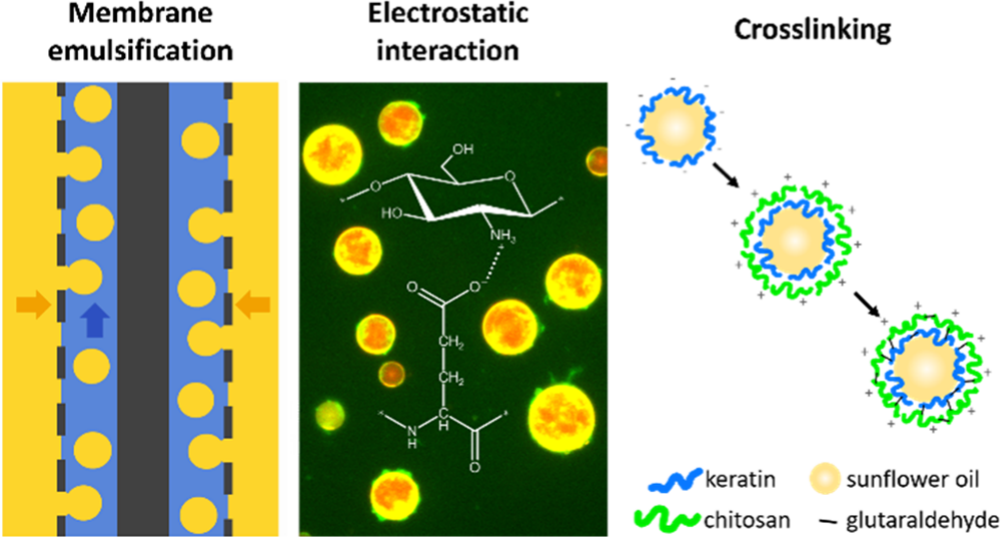

The continuous fabrication via membrane emulsification of stable microcapsules using renewable, biodegradable biopolymer wall materials keratin and chitosan is reported here for the first time. Microcapsule formation was based on opposite charge interactions between keratin and chitosan, which formed polyelectrolyte complexes when solutions were mixed at pH 5.5. Interfacial complexation was induced by transfer of keratin-stabilized primary emulsion droplets to chitosan solution, where the deposition of chitosan around droplets formed a core–shell structure. Capsule formation was demonstrated both in batch and continuous systems, with the latter showing a productivity up to 4.5 million capsules per minute. Keratin–chitosan microcapsules (in the 30–120 μm range) released less encapsulated nile red than the keratin-only emulsion, whereas microcapsules cross-linked with glutaraldehyde were stable for at least 6 months, and a greater amount of cross-linker was associated with enhanced dye release under the application of force due to increased shell brittleness. In light of recent bans involving microplastics in cosmetics, applications may be found in skin-pH formulas for the protection of oils or oil-soluble compounds, with a possible mechanical rupture release mechanism (e.g., rubbing on skin).

## Abstract 2

Microcapsules of sunflower oil in sustainable biomaterials were made by low-energy membrane emulsification with keratin and interfacial deposition of oppositely charged chitosan.

## Introduction

Microencapsulated oils have a wide variety of applications across a range of industries, including food, household,^1^ cosmetic,^2^ and pharmaceutical^3^ products. Encapsulation within a polymeric shell not only allows their dispersal in a polar environment but also offers benefits such as protection from oxygen degradation,^4^ improved retention of volatile components,^1^ and controlled release of the contents.^3^ The diameter of microcapsules can range between 1 μm and a few mm,^5^ making them small enough to pass through wastewater treatment plants into aquatic environments,^6^ contributing to microplastic pollution when synthetic and non-biodegradable wall materials are used (e.g., polyethylene glycol,^7^ polymethyl methacrylate,^8^ or melamine-formaldehyde^1^). The environment is polluted with 36,000 tons of microplastics each year in the EU alone^9^ and concerns over the implications for aquatic life and human health have grown with the emergence of studies confirming the presence of microplastics in the entire human food supply chain.^10^

While steps have been made to tackle microplastic pollution, including enacted and proposed limited bans on plastic microbeads,^9^ there remains a need to develop microcapsules based on biodegradable and non-toxic materials. Research on the use of biopolymers for microencapsulation is robust, with most investigated biopolymers including alginate, casein, whey proteins, chitosan, soy proteins, gluten, silk fibroin, zein, starch, and cellulose.^11^

Oppositely charged biopolymers can form complexes with each other via attractive electrostatic forces,^12^ and this mechanism is utilized in coacervation-based microencapsulation techniques such as complex coacervation^13^ and layer-by-layer methods.^2^

Being non-toxic, renewable, and biodegradable, the wall materials used for microencapsulation should be inexpensive and abundant, ideally existing in underutilized industrial waste streams. Keratin, a structural animal protein, meets all of the above requirements, with millions of tons of unutilized keratinous waste produced each year.^14^ Keratin can be solubilized from waste wool or feathers by sulfitolysis, reduction, or other methods,^15^ is negatively charged over a range of pH values,^16^ and has surface-active and emulsifying properties.^17^ Keratin has been used as a building block in the synthesis of multilayer films of alternating anionic keratin and a cationic polyelectrolyte;^16^ however, no examples were found in the literature of keratin being used in coacervation or layer-by-layer style microencapsulation of a liquid core.

Chitosan is the second most abundant biopolymer on the planet after cellulose, obtained from crustacean waste by deacetylation of chitin,^18^ most of which has no downstream use,^19^ making it another ideal sustainable biomaterial. Critically, chitosan is positively charged below its p*K*_a_ (∼6.5)^20^ and, therefore, complexation with keratin via electrostatic interactions is likely. Chitosan and keratin have been previously combined to prepare composite films,^21^ and chitosan has been used in conjunction with other anionic biopolymers in similar microencapsulation systems.^13,22^

Most instances of coacervate-based microcapsules in the literature use homogenization as the method of primary emulsification; however, the utilization of membrane emulsification (ME) can offer several advantages.^23^ In ME, the disperse phase (DP) is injected through a porous membrane into the continuous phase (CP) where droplet detachment is driven by shear stress across the membrane surface. The size of the droplets can be tuned by careful control of the process parameters, resulting in the production of monodisperse emulsions.^24^ Due to the low energy of ME however, the kinetics of adsorption of an emulsifier at the emerging oil–water (O/W) interface is critical for the production of stable emulsions with narrow droplet size distributions.^25^ While soluble keratin has been reported to produce stable emulsions by ultrasonication,^17^ the use of keratin in ME has not previously been attempted to the authors’ knowledge.

In the present study, the formation of stable microcapsules based on the electrostatic interactions between keratin and chitosan is reported for the first time. ME was utilized to generate the primary emulsion, in both batch and continuous configurations. Subsequently, the production of microcapsules from the primary emulsion was obtained by adsorption of chitosan to oppositely charged keratin at the droplet surface and cross-linking with glutaraldehyde (GTA). The properties and characteristics of the microcapsules and shell were examined by microscopy, zeta potential, and stability. Release studies were then carried out to assess the effect of chitosan absorption and cross-linking in the shell on the release of an oil-soluble dye from the encapsulated oil phase.

## Experimental Section

### Materials

Clean sheep’s wool was obtained from Wingham Wool Work. Sunflower oil was obtained from Tesco and used as the DP for the primary emulsion. Urea ≥ 98%, sodium metabisulfite ≥ 99%, tris(hydromethyl)aminomethane ≥ 99.8%, sodium dodecyl sulfate (SDS) ≥ 95%, hydrochloric acid (HCl, 35%), and sodium hydroxide (NaOH, 98%) were purchased from Fisher Scientific, UK. HCl and NaOH were diluted to 0.1 M as stock solution for pH adjustments; low-molecular-weight chitosan, acetic acid ≥ 99%, fluorescein isothiocyanate (FITC) ≥ 90%, methanol ≥ 99.9%, nile red ≥ 98%, hydrochloric acid 32%, and GTA solution grade II 25 wt % in H_2_O were obtained from Sigma-Aldrich UK and used without further purification.

### Preparation of Biopolymer Solutions

Keratin was extracted from wool using sodium metabisulfite as a reducing agent to cleave disulfide bonds.^15^ Clean sheep’s wool (30 g) was heated in 1 L of deionized water containing 8 M urea, 0.5 M sodium metabisulfite, 0.2 M tris base, and 0.2 M SDS (pH 7, adjusted using NaOH) at 65 °C for 5 h. The resulting aqueous extract was passed through a 50 μm mesh sieve and dialyzed against deionized water for 6 days using a cellulose tube membrane (MWCO 8 kDa), replacing the water daily. The solution was then diluted to 1 wt % concentration with deionized water, where the initial concentration of keratin was determined by the loss on drying method. For the loss on drying method, approximately 5 g of the sample was dried at 50 °C until no further change in mass was noted, and the mass of residual solids was calculated as a percentage of the initial sample mass.

Chitosan (1 wt %) was solubilized in 1 wt % acetic acid by overnight stirring at room temperature. The solution was vacuum filtered (Whatman, Grade 1), diluted to the desired concentration with deionized water, and adjusted to pH 5.5 using NaOH.

### Zeta Potential of Keratin Solution

The prepared keratin solution, to be used as the CP of the primary emulsion, was adjusted to pH values between 2 and 12 using NaOH and HCl. Each sample was loaded into a folded capillary cell, and the zeta potential was measured using a Zetasizer Nano ZSP instrument (Malvern Instruments, Malvern, UK). Two samples were prepared for each pH value, each measured in triplicate.

### Turbidity Measurement

Mixtures of keratin and chitosan solutions were prepared with a final concentration of 0.3 wt % chitosan and a range of keratin concentrations (0, 0.01, 0.05, 0.1, 0.2, 0.3, or 0.5 wt %). After stirring for 20 min at room temperature, the samples were diluted 10× with deionized water, and the transmission at 300 nm was measured using a Jenway UV–vis spectrophotometer (Cole-Palmer, St Neots, UK). Turbidity was calculated by subtraction of % trans from 100.

### Viscosity Measurement

The viscosity of the 1 wt % keratin solution and sunflower oil was measured using a Discovery HR-3 rheometer (TA Instruments, New Castle, USA). A shear rate sweep was conducted at 25 °C from 0.1 to 1000 1/s using a 40 mm cone (angle = 1°:0 min:25 s) and plate (gap = 29 μm).

### Interfacial Tension Measurement

The interfacial tension between the 1 wt % keratin solution and sunflower oil at 25 °C was measured using a FTA1000 B Class tensiometer (First Ten Angstroms, Portsmouth, USA) by the rising drop method. The sunflower oil DP was extruded from a hooked needle into the 1 wt % keratin CP, and the surface tension was determined from the shape of the rising drop before droplet detachment. An average of three measurements was taken (drop volume ∼4 μL).

### Stirred Cell Membrane Emulsification

O/W emulsions were prepared by stirred cell membrane emulsification (SCME) using a liquid dispersion cell (Micropore Technologies) and ringed, stainless-steel (SS), disc membranes. Prior to use in ME, the SS membranes (both disc and tubular) and additional inner rod underwent a standard cleaning procedure.^26^ Briefly, the items were immersed sequentially in an ultrasonic bath for 1 min in deionized water, 4 M NaOH, deionized water, 10 % wt citric acid, and finally deionized water. The items were soaked for 10 min in the acid and base solutions after sonication and were rinsed with tap water afterward, before being transferred to deionized water.

Sunflower oil (10 mL) was introduced using a syringe pump through the pores of the membrane into the cell containing 90 mL of keratin solution, where droplet detachment was facilitated by the wall shear generated from the paddle stirrer.

Using DOE software (MODDE Pro 12.1), a fractional experimental design with a linear model was implemented to explore the size and span (as responses) of emulsions generated using the dispersion cell. Three controllable emulsification parameters (pore size, stirring speed, and injection rate) were investigated as factors. The diameter of the pores was either 10 or 30 μm, while the stirring speed and injection rate ranged from 400 to 1100 rpm and 0.3 to 0.5 mL/min, respectively. 12 experiments were conducted including four center points (three repeats).

### Crossflow Membrane Emulsification (xME)

**Figure 1:**
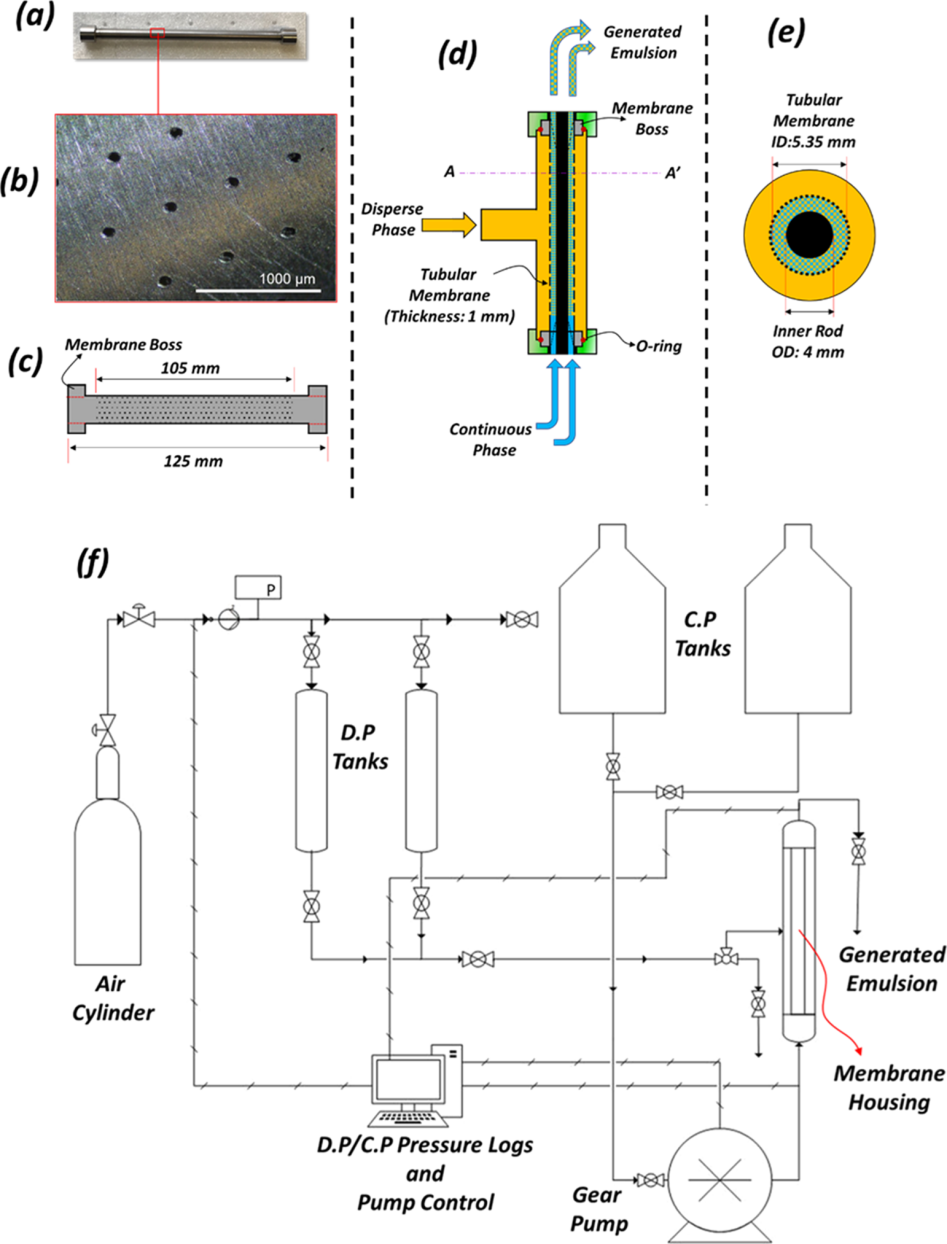
SS tubular membrane and assembly in membrane housing the (a) tubular membrane; (b) optical micrograph of tubular membrane pores; (c) schematic showing tubular membrane, its dimensions and boss positions; (d) schematic showing tubular membrane assembly in membrane housing; (e) schematic showing cross-section of A–A′ on membrane assembly; and (f) process flow diagram for the continuous crossflow ME apparatus used.

A bespoke system was designed and commissioned, consisting of a SS tubular membrane and its assembly in the membrane housing (Figure 1).

The SS tubular membrane and inner rod were obtained from Microkerf, Leicester, UK. The membrane (Figure 1a–c) was fabricated from a stainless tube with ID 5.35 mm and 30 μm pores (Figure 1b) laser drilled to cover the middle 105 mm of its 125 mm length with a 500 μm pitch.

The SS tubular membrane was cleaned, as described for the disc membrane before assembly in the membrane housing (Atech Innovations, Germany). For assembly into the housing (Figure 1d), the inner rod 4 mm OD was inserted into the SS membrane’s lumen and held into place by supports with drilled slits to allow for the crossflow of the CP within the created annulus (Figure 1d) bounded by the inner wall of the SS membrane and outer wall of the inner rod (4 mm). The membrane housing (and assembled components) was then attached to the continuous crossflow ME rig (Figure 1f). The CP and DP were pumped to the SS membrane housing at predetermined flowrates (via gear pump) and pressures (via compressed air), respectively, for crossflow droplet generation. The DP pressures, CP flowrates, and resultant transmembrane pressures for each droplet generation sample were acquired and logged using LabVIEW (National Instruments).

### Microcapsule Preparation

Primary emulsion droplets were isolated from the keratin solution by gravitational creaming in the absence of coalescence, and 1 mL of creamed droplets was mixed with 1 mL of deionized water and immediately added to 10 mL of 0.25 wt % chitosan. This was followed by the addition of 0, 25, or 50 μL GTA solution under stirring at room temperature. Samples were placed on a roller for 1 h and subsequently stored at room temperature.

### Imaging and Sizing of Emulsion and Microcapsules

Optical micrographs were captured using a SP400 microscope and digital camera (Olympus). Volume-weighted particle size distributions were obtained using a Mastersizer 3000 particle size analyzer and wet dispersion unit (Malvern Instruments) operating at 2000 rpm. The *D*_50_ and span were recorded.

### Monitoring of Adsorption of Chitosan

The zeta potential of primary emulsion droplets was measured before and after addition of the creamed droplets to chitosan solutions of different concentrations (0.001, 0.005, 0.01, 0.05, 0.1, 0.5, or 1 wt %) to monitor the adsorption of chitosan at the droplet surface. A washing step with deionized water was included before and after stirring in chitosan solution to remove excess polyelectrolyte. The zeta potential was measured, as described for the keratin solution.

### Fluorescence Microscopy

Fluorescently labelled chitosan was prepared by addition of 100 mg of FITC in 100 mL of methanol to 100 mL of 1 wt % chitosan solution and stirred overnight in the dark at room temperature.^27^ The chitosan was precipitated with NaOH, and unreacted FITC was removed by centrifugation (8000*g*, 10 min). The precipitate was washed with deionized water until the supernatant showed no fluorescence. The FITC-labelled chitosan was dissolved in 1 wt % acetic acid solution and dialyzed against deionized water for 3 days in the dark, replacing the water daily. The concentration of chitosan in the final solution was determined by the loss on drying method, and the solution was diluted with deionized water to 0.25 wt %. The pH was adjusted to 5.5 using NaOH.

Fluorescence micrographs were captured using an EVOS M5000 Imaging System (Thermo Fisher Scientific) fitted with a green fluorescent protein light cube with excitation (λ_ex_) and emission wavelengths (λ_em_) of 470 and 525 nm, respectively, for the visualization of FITC-labelled chitosan, and a red fluorescent protein light cube (λ_ex_ = 531 nm, λ_em_ = 595 nm) for the visualization of the nile red-stained oil, respectively. Prior to imaging, the microcapsules were dispersed in deionized water to reduce background fluorescence from unadsorbed chitosan.

### Release of Encapsulated Nile Red

Both uncross-linked and cross-linked microcapsules were prepared using sunflower oil stained with nile red (1 mg/mL) to make the primary emulsion. As a control, primary emulsion controls were prepared by mixing 1 mL of creamed droplets with 1 mL of deionized water and addition to 10 mL of 1 wt % keratin solution to ensure the same degree of dilution of the primary emulsion droplet suspension in all samples. Unstained sunflower oil (5 mL) was gently placed on top of each sample using an automatic pipette. The samples were either left static at room temperature for 5 days or centrifuged immediately (15 m, 5000×*g*). An aliquot (1 mL) was taken from the center of the oil layer, and the absorbance was measured at 520 nm by UV–vis spectrophotometry. An average result was taken from three repeats. A standard curve was prepared by the measurement of known concentrations of nile red-stained sunflower oil, diluted with unstained oil.

## Results and Discussion

### Complexation between Keratin and Chitosan

**Figure 2:**
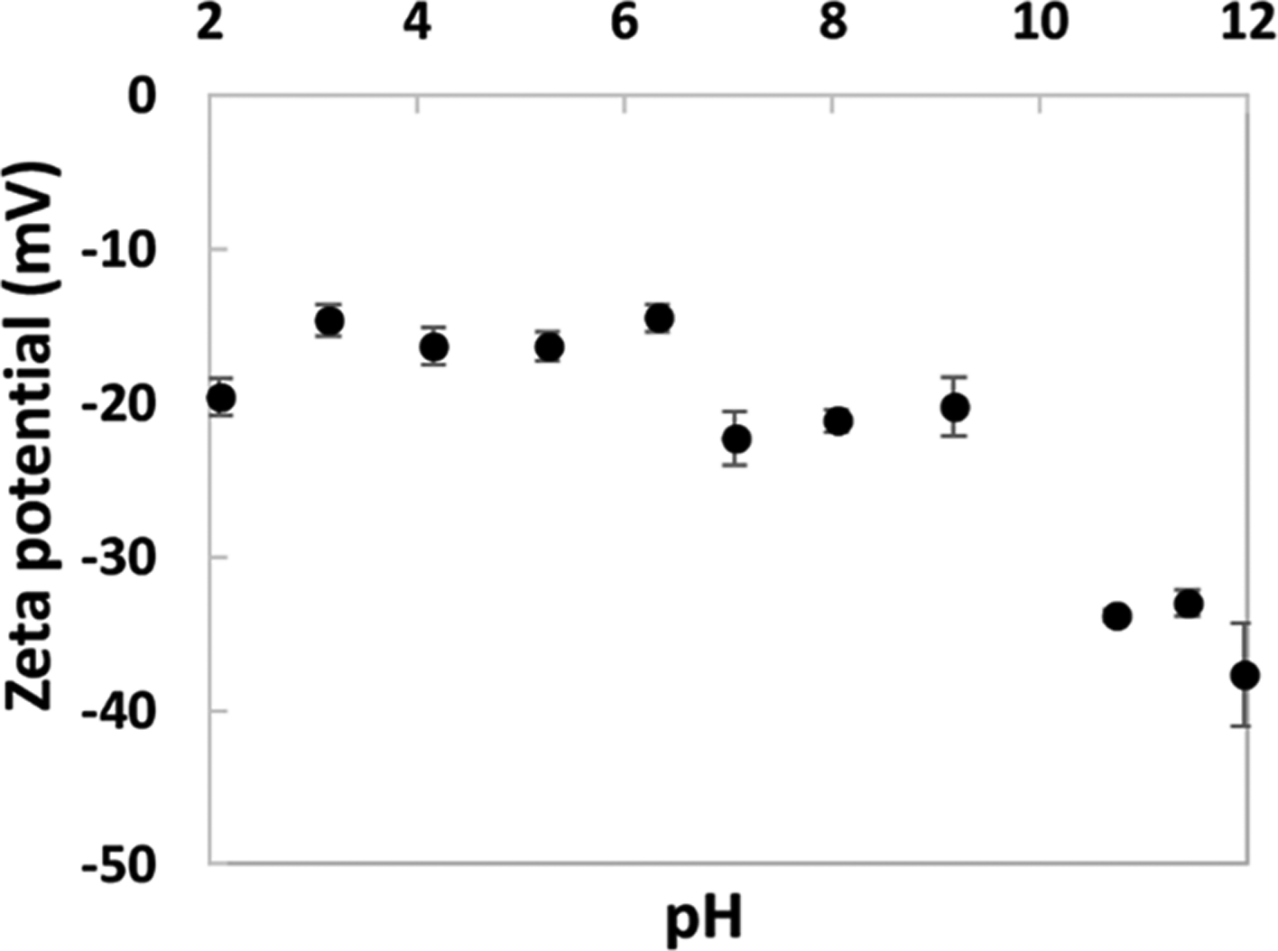
Zeta potential of keratin solution as a function of pH. Error bars represent standard deviation from three measurements.

The zeta potential of the extracted keratin between pH 2 and 12 was negative, with the magnitude of the net surface charge increasing with alkalinity (Figure 2) due to deprotonation of its amino groups. The values reported here are more negative than those reported in the literature,^16,28^ attributed to the use of the anionic surfactant SDS in the extraction process, included to prevent the major aggregation of solubilized keratin during dialysis. Previous research on the use of SDS in the extraction of feather keratin suggests that while most of the SDS was removed by dialysis, some remained complexed to keratin molecules which would impart a more negative overall charge.^29^

Since the keratin was negatively charged, it was expected to interact with chitosan to form polyelectrolyte complexes by opposite charge interactions at an appropriate pH below chitosan’s p*K*_a_ (∼6.5).^30^ Since the magnitude of the charge on the keratin decreased with increasing acidity, pH 5.5 was selected to ensure both polyelectrolytes carried a moderate charge.

**Figure 3:**
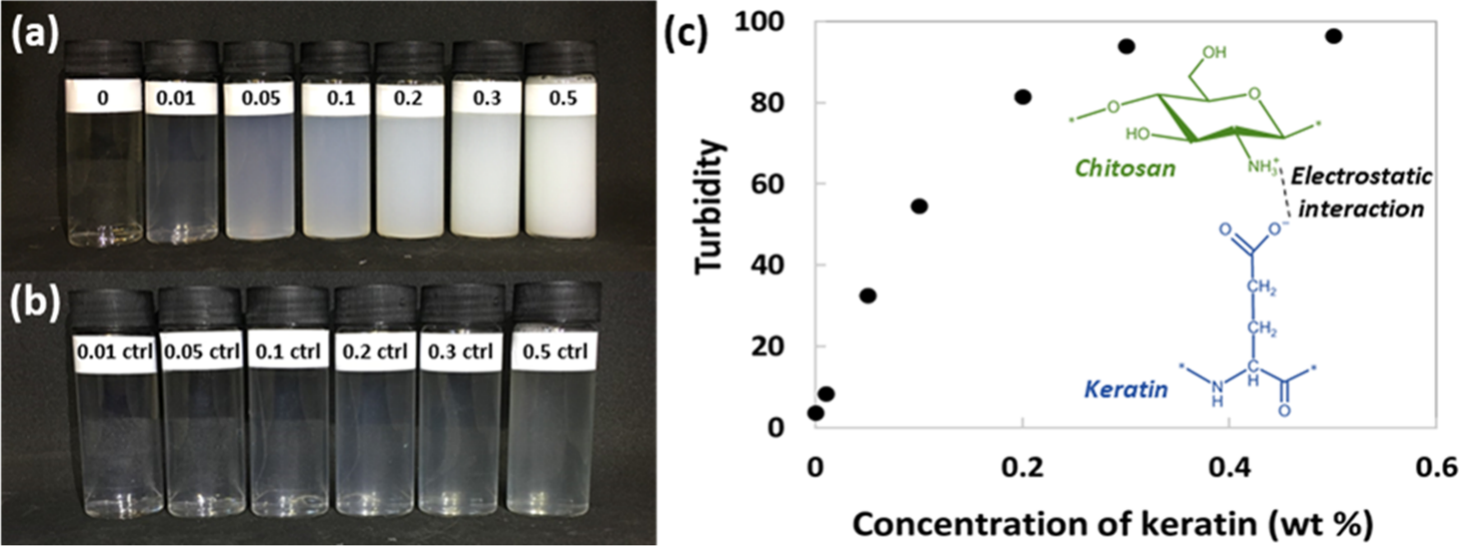
(a) Dispersions of mixed chitosan and keratin solutions (0.3 wt % chitosan, 0–0.5 wt % keratin, pH 5.5); (b) controls containing only keratin solution (0–0.5 wt % keratin, pH 5.5); (c) turbidity of dispersions containing 0.3 wt % chitosan and 0–0.5 wt % keratin, pH 5.5, diluted 10× with deionized water. Error bars (mostly smaller than the dot size) represent the standard deviation from three measurements. The diagram shows the proposed interaction mechanism of keratin and chitosan.

An opaque dispersion was observed when solutions of keratin and chitosan solution were mixed together at pH 5.5, indicating the formation of insoluble particles. The opacity of the dispersion became more pronounced with the increased keratin content (Figure 3a).

The degree of opacity was measured by turbidity quantification (Figure 3c). There was an initial rapid rise in turbidity with the increasing keratin content due to the increased presence of light-scattering polyanion–polycation complexes and then a levelling off at higher concentrations, which could be a result of multiple scattering effects due to a high concentration of particles or sedimentation of larger particles causing increased transmission of light through the sample. While complex assembly is thought to be driven mainly by the long-range electrostatic attraction between keratin’s negatively charged amino acid side chains and chitosan’s positively charged amino groups, medium-range hydrophobic interactions and short-range hydrogen bonding can also contribute to complex formation and stability.^12^ Wool keratin consists of a variety of amino acids with polar, non-polar, and ionizable side chains that allow for multiple interactions to take place.^31^ Both keratin and chitosan contain groups that can participate in hydrogen bonding, that is, chitosan’s hydroxyl groups and cysteine and serine in keratin, which contain a hydroxyl and sulfhydryl group, respectively. Although the deacetylated chitosan used in this work is hydrophilic in nature,^32^ hydrophobic interactions may take place between keratin’s non-polar amino groups (e.g., leucine and valine) and chitosan’s acetyl groups.

### Primary Emulsion Generation by Stirred Cell Membrane Emulsification

**Figure 4:**
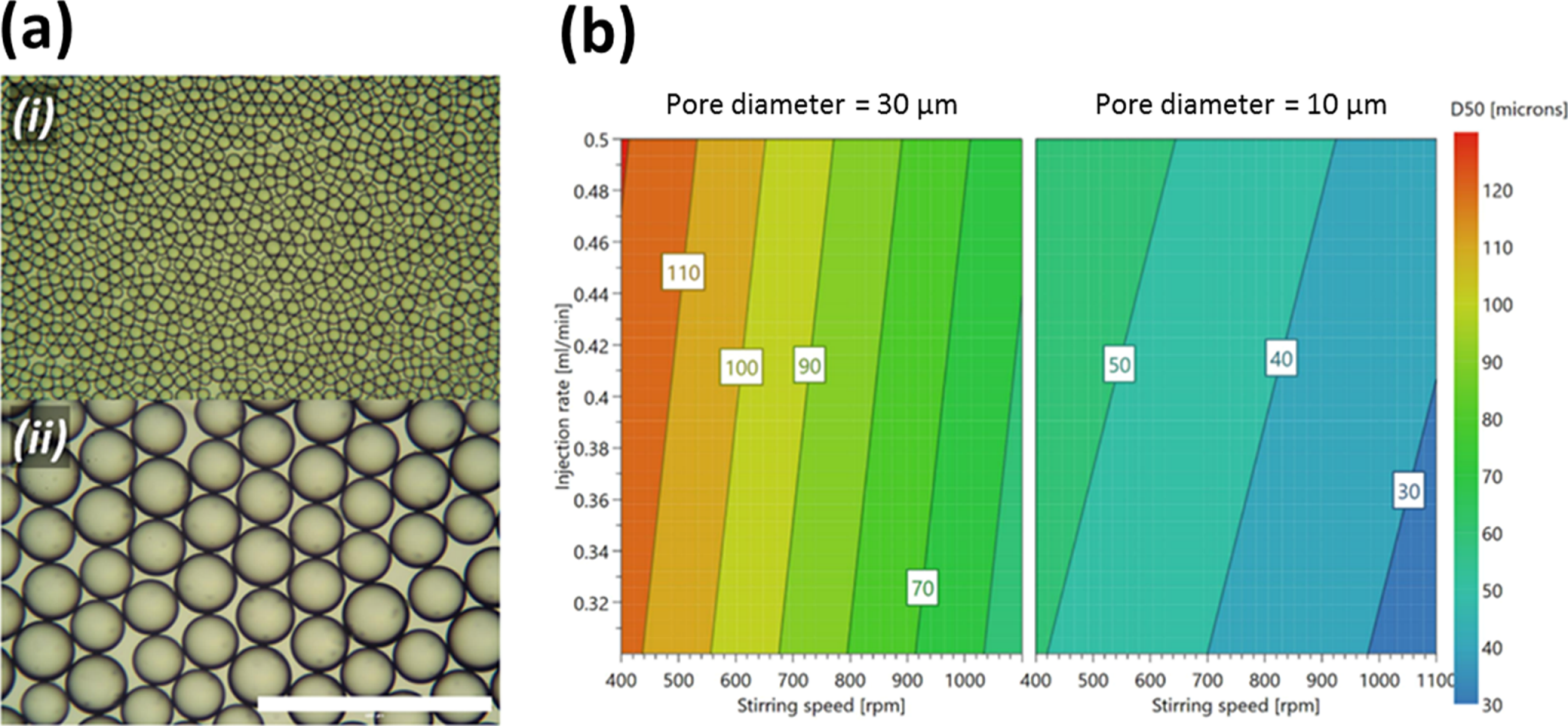
(a) Optical micrographs of smallest and largest keratin-stabilized microdroplets produced by stirred cell ME of sunflower oil in 1 wt % keratin solution: (i) experiment 3: *d*_p_ = 10 μm, injection rate = 0.3 mL/min, stirring speed = 1100 rpm, and *D*_50_ = 29.9 μm; (ii) experiment 6: *d*_p_ = 30 μm, injection rate = 0.5 mL/min, stirring speed = 400 rpm, and *D*_50_ = 126 μm. Scale bar = 500 μm. (b) 4D contour plots showing the predicted *D*_50_ (median volume diameter) of emulsions of sunflower oil in 1 wt % keratin solution produced by stirred cell ME.

After the confirmation of complexation between keratin and chitosan, the next step was to apply the interaction at the interface of an emulsion. The ME of the primary emulsion (stabilized by keratin) was explored by small batch (100 mL) SCME to scope the droplet size range and uniformity of generated emulsions prior to scaling up to continuous crossflow ME (xME). Table S1 summarizes the DOE and experimental data for the 12 experiments conducted. Droplets with median volume diameters (*D*_50_) between 30 and 126 μm (Figure 4a) were generated using a membrane pore diameter of either 10 or 30 μm and varying the injection rate and stirring speeds between 0.2 and 0.5 mL/min, and 400–1100 rpm, respectively. Results from the DOE showed a good fit and future prediction precision of *R*^2^ = 0.99 and *Q*^2^ = 0.87 for the *D*_50_ (Table S2), which allowed the estimation of *D*_50_ at any given space within the range of parameters tested (Figure 4b). This was confirmed by validation experiments carried out with both membrane pore sizes investigated, with excellent results (Table S3).

The *D*_50_ was dependent on all factors included in the DOE, with pore size having the greatest influence, followed by stirring speed (Table S2). The size of the pores is a major factor in determining the size of the droplets produced by ME, with droplets produced here being 2–6 times larger than the pore diameter, in agreement with the 2–10 ratio found in the literature.^33^ The stirring speed had a strong influence on droplet diameter as it generated the shear which causes droplet detachment.^34^ Stirring speeds between 400 and 1100 rpm enabled the controlled access to a wider range of droplet size categories for the 30 μm pore size than the 10 μm pore size (Figure 4b). Although a higher injection rate results in a greater volume of DP permeated through the membrane before droplet detachment and, hence, in larger droplets,^34^ its effect in the design space used here was minimal compared to other factors (Table S2). This result also implies the absence of any transition from dripping to jetting regimes or vice versa, which would have resulted in a clear discontinuity in droplet diameter.

The span, a dimensionless number indicating the width of the distribution of the emulsions, ranged from 0.368 to 0.923 (Table S1). The DOE was used to identify the parameters where span would be lowest, and therefore, the droplets would be most uniform. The model was tuned in order to improve the fit and future prediction precision by log transformation, removal of an insignificant term (injection rate), and addition of an identified squared term (stirring speed), resulting in an *R*^2^ value of 0.95 and *Q*^2^ value of 0.79. For the 30 μm pore diameter, DOE results indicated that low stirring speeds promoted monodispersity. Within the design space, droplets generated at 400 rpm were, therefore, most uniform. An opposite effect was observed with the 10 μm membrane whose uniformity increased slightly with increasing stirring speed. The impact of stirring speed was more significant when using the larger pore size and when the stirring speed was higher. It was concluded therefore that droplet breakup at high shear was responsible for the relatively poor span seen in some samples from membranes with a larger pore size, and the lower predictability of the span model versus the D_50_ model, and hence the minor upper limit deviation of 3.0 and 3.5% for the 10 and 30 μm pore membranes, respectively, in span validation experiments (Table S3).

### Scale-Up with Crossflow Membrane Emulsification

Using as a starting point the conditions which gave the lowest span in the stirred cell setup (experiment 6, *D*_50_ of 126 μm, span = 0.368, with a 30 μm pore membrane), the wall shear (τ_SMCE_) of 2.043 Pa was approximated using eqs S3–S6 for the xME equipment design values of impeller diameter (*D*) = 0.03 m, tank diameter (*T*) = 0.035 m, blade height (*b*) = 0.011 m, number of blades (*n*_b_) = 2; membrane morphology values of *r*_1_ and *r*_2_ of 0.008 and 0.011 mm as the respective outer and inner radii of the porous region of the ringed membrane; CP properties μ_c_ and ρ_c_ of 0.00101 Pa s and 1000 kg/m^3^, respectively; and emulsification ω of ≈41.9 s^–1^ @ 400 rpm.

**Figure 5:**
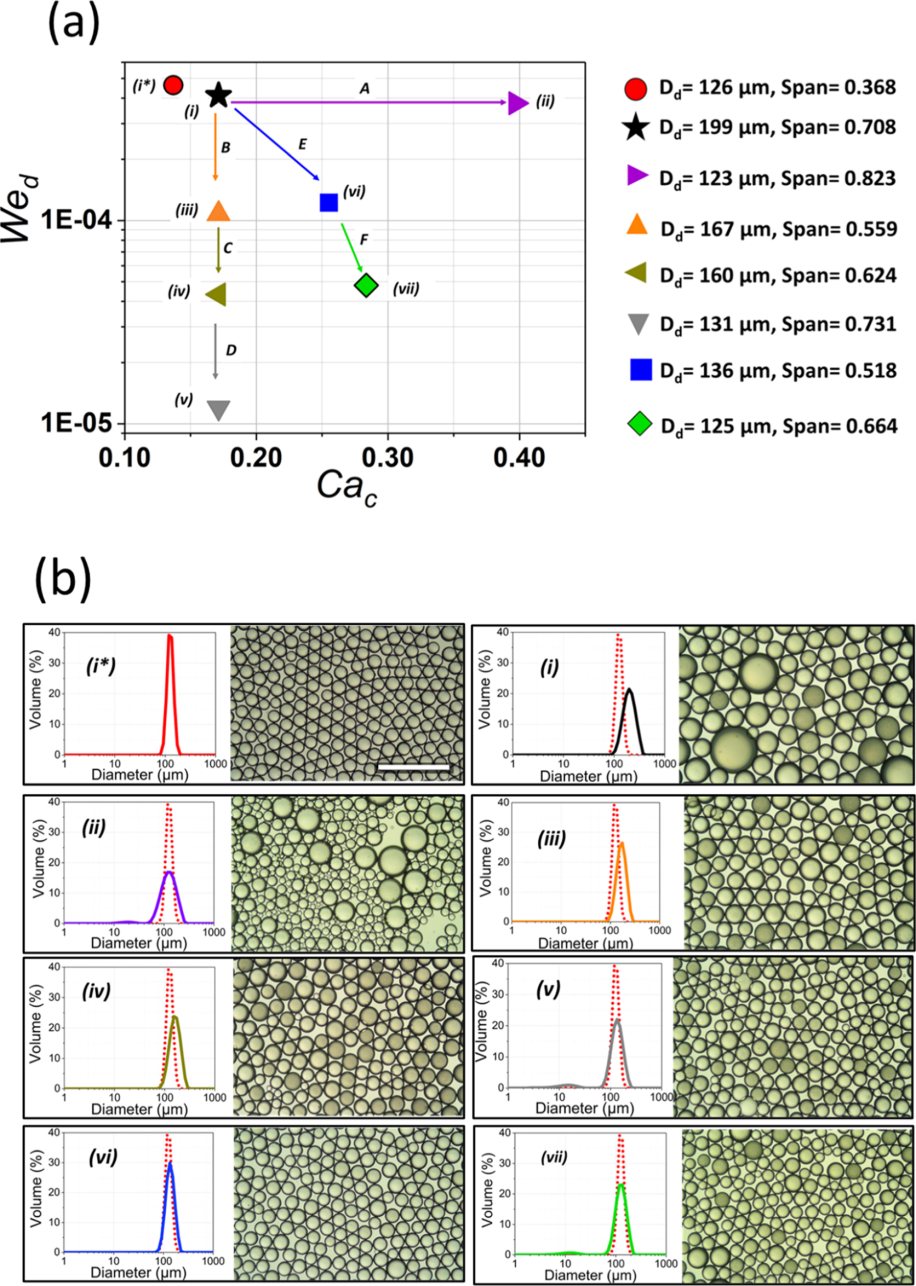
(a) We_d_–Ca_c_ plot showing keratin-stabilized microdroplets produced using the SCME and crossflow ME rig. For XME, path A shows Ca_c_ increase, paths B, C, and D show We_d_ reduction, while paths E and F show simultaneous increase in Ca_c_ and reduction in We_d_; (b) size distributions and optical micrographs of keratin-stabilized microdroplets produced using (i*) SCME (experiment 6: *D*_50_ = 126 μm, span = 0.368) and (i–viii) xME; insets show particle size distributions, using (i*) as a reference in all distributions. For (a,b), (i) *D*_d_ = 199 μm, span = 0.708; (ii) *D*_d_ = 123 μm, span = 0.823; *D*_d_ = 167 μm, span = 0.559; *D*_d_ = 160 μm, span = 0.624; *D*_d_ = 131 μm, span = 0.731; *D*_d_ = 136 μm, span = 0.518; and *D*_d_ = 125 μm, span = 0.664.

This resulted in significantly larger droplets, with *D*_50_ = 199 μm and a span = 0.708, for approximate DP Weber number (We_d_) of 4.1 × 10^–4^ and CP capillary number (Ca_c_) of 0.171, respectively (Figure 5i), evaluated using eqs S1 and S2. As the membrane in the xME configuration has approximately 10× more pores than the SCME disc membrane, owing to the increased pore area of the membrane, a proportionally higher DP flowrate was needed to maintain the same We_d_ (∼5 mL/min of sunflower oil in the xME system compared to 0.5 mL/min in the SCME). The CP flowrate needed to obtain similar shear, approximated by equation S7, was applied to the xME (i.e., 300 mL/min).

From this first value, the xME system was further tuned following three strategies (Figure 5a): increasing Ca_c_ at constant We_d_; reducing We_d_ at constant Ca_c_; and a combination of increasing Ca_c_ and reducing We_d_.

For the first strategy, droplet diameters with *D*_50_ approaching the values in the SCME were obtained by increasing the shear of the xME system to Ca_c_ values of ≈0.393 (700 mL/min) from 0.171 at nearly constant We_d_ (Figure 5a, path A), which resulted in droplets generated with a *D*_50_ of 123 μm but with a higher span of 0.892 (Figure 5ii) and ∼4× higher droplet throughout. The higher span and broadening of the droplet size distribution (cfr. Figure 5ii) of a narrowing jetting regime are characterized by jet breakup at multiple points of the dispersed phase jet.^35^

For the second strategy, the We_d_ was reduced ∼4× to 1.1 × 10^–4^ (cfr. Figure 5a, path B) at constant Ca_c_ (0.171), leading to an increased diffusion of keratin from the bulk CP to the interface and, consequently, promoting droplet stability due to a slower dispersed phase droplet growth. However, further We_d_ reduction to 4.3 × 10^–5^ (Figure 5, path C) resulted in droplets with a *D*_50_ reduction from 167 to 160 μm but an increased span from 0.559 to 0.624. Continuous We_d_ reduction from 4.3 × 10^–5^ (Figure 5iv) to 1.2 × 10^–5^ (Figure 5v) resulted in progressively smaller yet less uniform droplets (Figure 5a, path D). This reduced uniformity with reducing DP inertia is due to, again, an onset of thinning jetting, as evident from the increased number of small droplets (Figure 5v).

In both cases of thinning jetting (i.e., Figure 5ii,v), larger droplets were observed in the extreme of point (ii) as a result of poor keratin interface saturation of small microdroplets formed at the inception of jetting, with a large surface area that are not properly coated with keratin which coalescence to form the larger droplets. This occurred less in point (v) due to lower We_d_ (hence, lower droplet generation frequencies) that enabled interface saturation at the inception of jetting. The formation of large droplets at high shear is seldom observed in surfactant systems due to the smaller molecular size of surfactants which promotes fast migration to the interface.^35^

Paths A, B, C, and D demonstrate droplet generation scenarios where increased Ca_c_ to We_d_ ratios were implemented to obtain droplets with *D*_50_ ≈ *D*_50,SCME_. For the third strategy, an increased Ca_c_ to We_d_ ratio was accomplished by a simultaneous increase in Ca_c_ and decrease in We_d_ (i.e., Figure 5, paths E and F). This was done just enough to reduce the droplet size to avoid thinning jetting. Point (vi) of Figure 5 depicts droplets formed at a Ca_c_ of 0.256 (450 mL/min) and We_d_ of 1.2 × 10^–4^ to obtain droplets with a *D*_50_ of 136 μm and span of 0.518 (Figure 5vi) that were more uniform than points (i,ii). Further simultaneous Ca_c_ increase with We_d_ reduction led to droplets possessing a *D*_50_ of 125 μm and span of 0.664 (Figure 5vii). This was carried out at a Ca_c_ of 0.280 (500 mL/min) and We_d_ of 4.8 × 10^–5^ (Figure 5vii).

This investigation, therefore, showed how the operational space of We_d_–Ca_c_ can be leveraged to strategically tune the properties of generated emulsions with the xME.

### Microcapsule Formation and Stability

**Figure 6:**
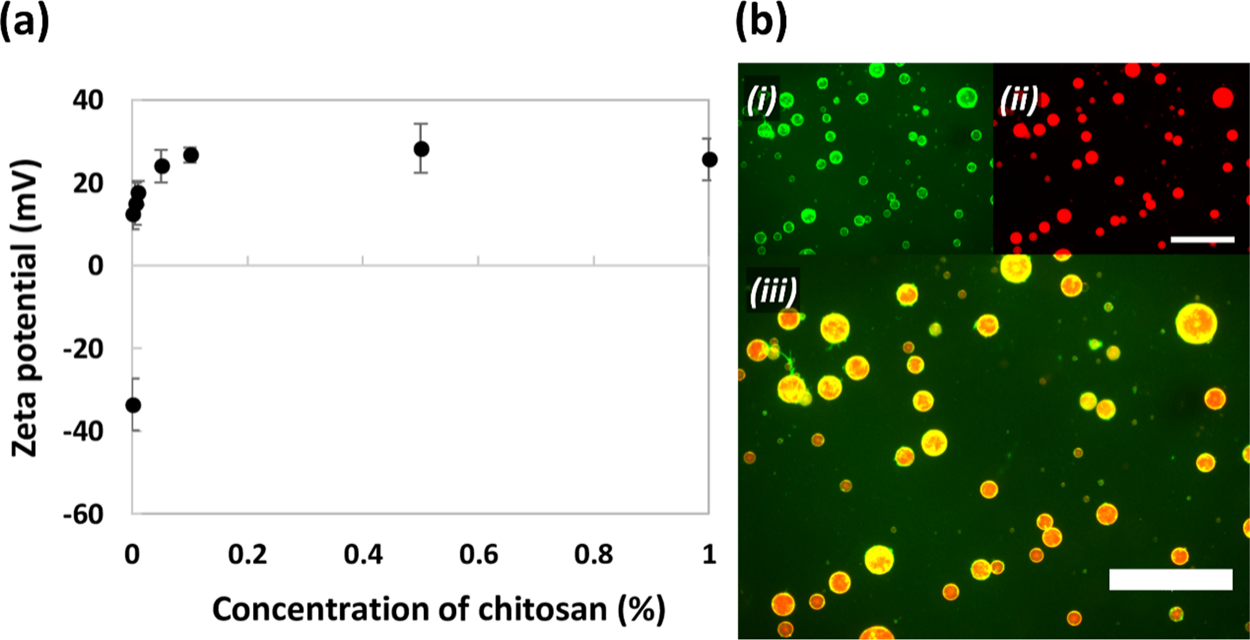
(a) Zeta potential of keratin-stabilized oil droplets after treatment with 0–1 wt % chitosan solution. Error bars represent standard deviation from three measurements. (b) Fluorescence microscopy images of keratin–chitosan microcapsules containing sunflower oil. (i) FITC-labeled chitosan (λ_ex_ = 470 nm, λ_em_ = 525 nm); (ii) nile red-stained sunflower oil (λ_ex_ = 531 nm, λ_em_ = 595 nm); and (iii) merged. Scale bars = 750 μm.

Zeta potential measurements were used to monitor the deposition of chitosan at the surface of keratin-stabilized emulsion droplets to form the microcapsule shell. The untreated primary emulsion droplets had a negative zeta potential (between −20 and −30 mV) due to the negatively charged keratin at the interface (Figure 6a). After treatment with chitosan, charge reversal occurred, indicating the adsorption of positively charged chitosan at the droplet surface with the zeta potential increasing sharply with increasing concentration of chitosan and then leveling off at 20–30 mV, suggesting adsorption saturation. As such, a concentration of 0.25 wt % chitosan was chosen as the optimal value.

The microcapsule structure was visualized by fluorescence microscopy of samples made with FITC-labeled chitosan and nile red-stained sunflower oil. The location of FITC-chitosan, after removal of excess from the CP by dilution in water, was concentrated at the droplet surface (Figure 6bi), and the location of the oil phase was confirmed inside the microcapsules (Figure 6bii). Both images merged together (Figure 6biii) demonstrate a core–shell structure, confirming the zeta potential results.

The attraction between biopolymers within a polyelectrolyte complex differs in strength depending on the characteristics of the biopolymers in question and the environmental conditions,^12^ and coacervate microcapsules sometimes require chemical cross-linking to give strength and stability to the shell.^36^ Therefore, different quantities of GTA solution were added during microcapsule formation to cross-link between the amino groups of keratin and chitosan molecules.

**Figure 7:**
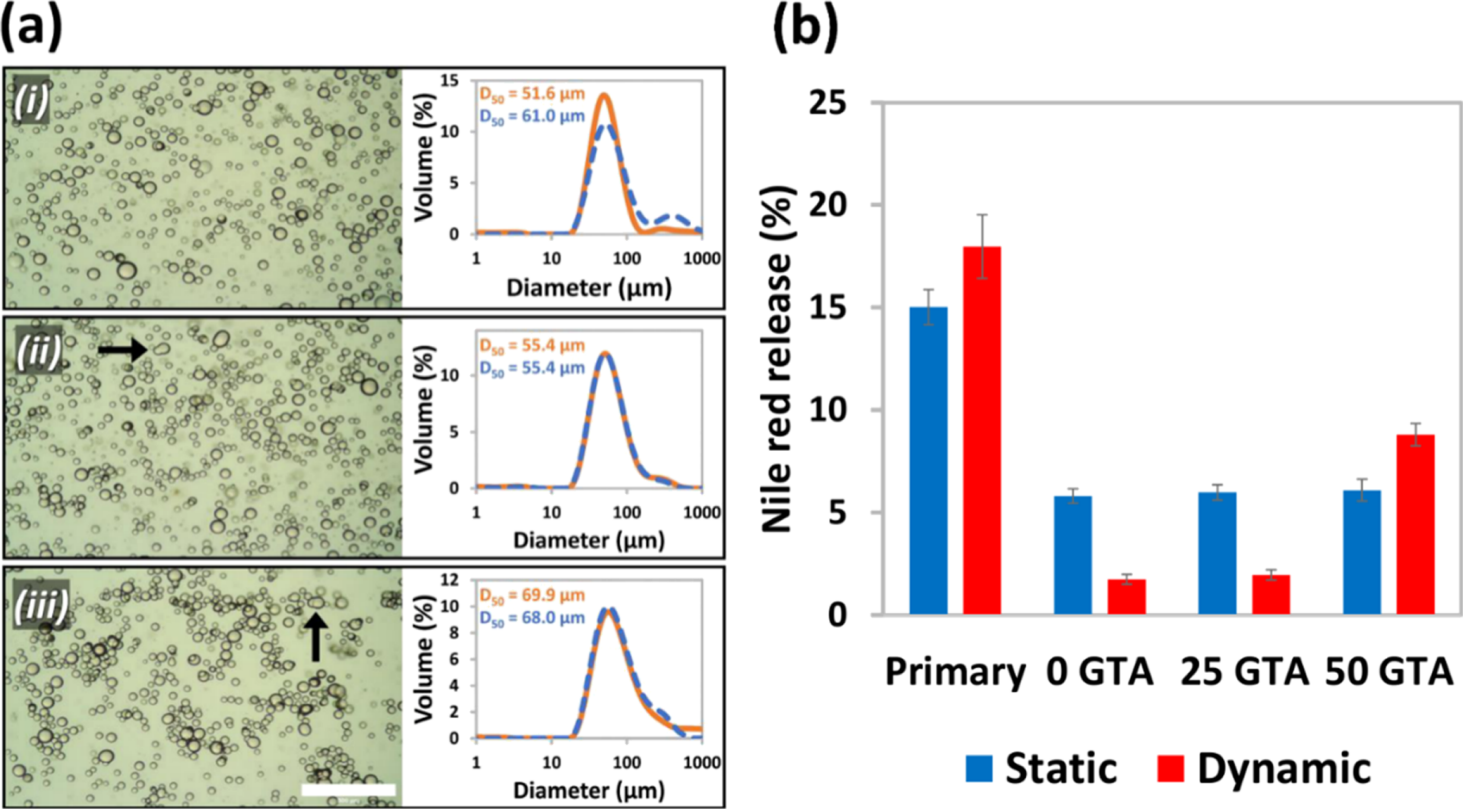
(a) Optical micrographs and particle size distributions of keratin–chitosan microcapsules: (i) uncross-linked (ii) cross-linked by addition of 25 μL GTA solution per 10 mL sample; (iii) cross-linked by addition of 50 μL GTA solution per 10 mL sample, immediately after synthesis (orange solid line) and after 6 months of ambient storage (blue dashed line). Scale bar = 500 μm. Arrows point to irregular structures. (b) Percentage release of nile red from primary emulsion droplets stabilized by keratin alone versus keratin–chitosan microcapsules cross-linked with 0–50 μL of GTA solution per 10 mL of the sample, after 5 days static incubation at room temperature, or 15 min centrifugation (5000*g*). Error bars represent standard deviation from three measurements.

The stability of the cross-linked and uncross-linked microcapsules was assessed in terms of both size and integrity. For the former, storage for 6 months resulted in no significant change to the size distribution or *D*_50_ of the cross-linked microcapsules (Figures 7ai,iii and S1), whereas a significant increase in average particle size was observed in the uncross-linked sample (Figure 7ai), suggesting that cross-linking enhances long-term stability. GTA addition increased the initial *D*_50_ due to cross-linking of microcapsules into clusters, which the laser cannot distinguish from a single particle, hence the greater variability in results for the most highly cross-linked sample containing 50 μL of GTA solution.

The stability of the microcapsules was further investigated by studying the release of an encapsulated dye from the microcapsules into an external free oil phase under both static and dynamic conditions. After 5 days of static incubation at room temperature, significantly less nile red was released from the microcapsules as compared with the primary emulsion (Figure 7b), probably due to a gel-like coacervate network at the interface, which is thought to reduce permeability to small molecules.^37^ Upon applying centrifugal force, while all microcapsule samples released less dye than the primary emulsion, the percentage nile red release from microcapsules cross-linked with 50 μL of GTA was around 10 times higher than uncross-linked microcapsules or those cross-linked with 25 μL of GTA. This was attributed to a more rigid, brittle shell caused by a high number of covalent cross-links between biopolymer molecules,^38^ making the most highly cross-linked capsules more susceptible to breakage under the application of force. The observation of non-spherical microcapsules only in samples treated with GTA (Figure 7a arrows) supports the view of reduced elasticity and fluidity of the interfacial membrane as a result of cross-linking. These characteristics could be tailored by changing the cross-linker, for example, using genipin, a plant-sourced cross-linking agent.^39^

### Productivity and Scale-Up

The concentration and frequency of generated emulsions are important indices in determining the ideal emulsification conditions for scale-up. In the continuous xME, the generation of smaller droplets requires high CP flowrates, which results in a less concentrated emulsion. The use of an inner rod alleviates this problem,^40^ with a 79% increase in the droplet concentration compared to the case without a rod in the present work. To obtain the same droplet concentration in a system without the inner rod, recirculation of the CP would be required to meet the high shear requirement, resulting in a multiple-pass system, with negative effects on emulsion quality and energy consumption.^6,41^

Scale-up with the continuous xME also showed increased droplet generation frequency, as compared to the batch SCME, leveraging an increased membrane surface area. Consequently, a higher DP flux and emulsion productivity were achieved. Considering the data, as shown in Figure 4, although *D*_50_ ≈ *D*_50,SCME_ for point (ii), a droplet generation frequency of 76,168 droplets/s (equivalent to about 4.5 million droplets per minute) was obtained due to the xME’s membrane pore area being ∼10 times that of the SCME for the same DP flux. Further increases in droplet generation, while maintaining emulsion quality, can be obtained by increasing the membrane diameter and/or reducing the pitch length between pores. For example, doubling the inner diameter of the membrane at constant annular diameter and membrane thickness, or doubling the length of the membrane would double the frequency of produced droplets produced at point (vii) conditions to ∼51,000 droplets/s. Reducing the pitch length by 50% would have the greatest productivity effect by increasing the droplet generation frequency 4-fold to ∼103,000 droplets/s. A combination of the three changes would result in a 16-fold higher droplet generation frequency. These values, together with numbering-up strategies, show that the keratin–chitosan microcapsules could be produced at the industrial scale.

## Conclusions

The production of stable microcapsules using renewable and biodegradable biopolymer wall materials, keratin and chitosan, is reported here for the first time. The compatibility and scale-up potential of the formulation were demonstrated with ME. Turbidity measurements confirmed the complexation of keratin and chitosan at pH 5.5 which were linked to electrostatic attraction arising from their opposite charges, and chitosan was seen to adsorb at the surface of keratin-stabilized primary emulsion droplets by zeta potential measurements and fluorescence microscopy. Using ME, it was possible to generate primary emulsion droplets with diameters of 30–126 μm and a span as low as 0.394.

Keratin–chitosan microcapsules cross-linked with GTA showed significant stability over time, with no increase in size after 6 months in storage under ambient conditions. Considering the non-toxicity and biocompatibility of keratin and chitosan, the stability of microcapsules at skin-pH, and the possible release mechanism of mechanical rupture (e.g., rubbing on skin), these capsules may find use in cosmetic, personal care, or biomedical products.

## Supporting Information Available

Stirred cell membrane emulsification parameters and droplet D50 and span; coefficients for SCME DOE models; shear stress approximations; and average particle size (D50 by volume) over time of keratin–chitosan microcapsules (PDF)

## Notes

The data set supporting this paper is openly available from the University of Bath data archive at https://researchdata.bath.ac.uk/id/eprint/966.
