# Supplementary material for: Keratin–Chitosan Microcapsules via Membrane Emulsification and Interfacial Complexation
Source: ACS Sustain Chem Eng. 2021 Dec 1;9(49):16617–26. doi: 10.1021/acssuschemeng.1c05304 (PMC8735752; doi:10.1021/acssuschemeng.1c05304)
Supplement: Supplementary file 1 — sc1c05304_si_001.pdf [file sc1c05304_si_001.pdf]

Supporting Information

Keratin-chitosan microcapsules via membrane emulsification and interfacial complexation

Amy Wilson,[†] Ekanem E Ekanem,[‡] Davide Mattia,[‡] Karen J Edler,[†] Janet L Scott[†]

[†]Department of Chemistry, University of Bath, Claverton Down, Bath, BA2 7AY, United Kingdom

[‡]Department of Chemical Engineering and Centre for Advanced Separations Engineering, University of Bath, Claverton Down, Bath, BA2 7AY, United Kingdom

Pages 5, Figures 1, Tables 2.

Section S1. Design of Experiments

Table S1. Stirred cell membrane emulsification parameters and droplet D_{50} and span.

Exp.	d_p (μm)[a]	Injection rate (mL/min)	Stirring speed (rpm)	D_{50} (μm)[b]	Span
1	10	0.3	400	52.6	0.541
2	30	0.3	400	109	0.394
3	10	0.3	1100	29.9	0.511
4	30	0.3	1100	57.8	0.738
5	10	0.5	400	62.2	0.575
6	30	0.5	400	126	0.368
7	10	0.5	1100	33.4	0.513
8	30	0.5	1100	59.6	0.923
9	10	0.4	750	38.1	0.591
10	10	0.4	750	40.5	0.568
11	10	0.4	750	39.4	0.599
12	30	0.4	750	85.9	0.634

[a]Pore diameter. [b]Median volume diameter.

Table S2. Coefficients for SCME DOE models.

Factor	Model 1 (D50)	Model 2 (Span)

	Coeff. SC	Std. Err.	P	Conf. int. (±)	Coeff. SC	Std. Err.	P	Conf. int. (±)
Constant	61.2	1.21905	3.26E-10	2.88262	-0.22009	0.014806	1.49E-06	0.035012
d_p (10 μm)	-23.459	1.27325	3.44E-07	3.0108	-0.01254	0.009015	0.206702	0.021317
d_p (30 μm)	23.459	1.27325	3.44E-07	3.0108	0.012544	0.009015	0.206702	0.021317
Stirring speed	-16.6353	1.29082	3.93E-06	3.05233	0.050216	0.008874	0.000768	0.020984
Stirring speed * Stirring speed	-	-	-	-	-0.03159	0.013316	0.049445	0.031487
Injection rate	3.41538	1.27325	0.03143	3.0108	-	-	-	-
d_p (10 μm) * Stirring speed	7.40073	1.31127	0.000779	3.1007	-0.08218	0.009015	3.92E-05	0.021317
d_p (30 μm) * Stirring speed	-7.40073	1.31127	0.000779	3.1007	0.082178	0.009015	3.92E-05	0.021317

d_p = pore diameter. Dashes indicate no data (term not included in model).

Section S2. Shear Stress Approximations

As with all membrane emulsification systems with surface shear, the diameter of the generated droplets is dependent on the combined competitive detachment effects (mainly due to the inertial and shear forces exerted by the dispersed phase and continuous phase, respectively) and retention effects (mainly the interfacial tension effect).[1] The ratio of these detachment effects to the interfacial tension force are represented by, respectively:[2]

$$We_d = \rho_d u_d^2 d_p / \gamma \quad (S1)$$

and

$$Ca_c = \mu_c u_c / \gamma \quad (S2)$$

Where We_d is the Webber number of the dispersed phase, ρ is the density of dispersed phase, u_d is the velocity of disperse phase at a pore entrance, d_p is the pore diameter of the membrane, Ca_c is the capillary number of the continuous phase, μ_c is the viscosity of the continuous phase, u_d is the velocity of continuous phase at the inlet to membrane and γ is the interfacial tension between disperse and continuous phases.

Amongst other factors such as formulation of phases, membrane morphological properties and membrane equipment design, the right balance between these forces (operational factors) determines the diameters and degree of uniformity of generated droplets to a large extent.[1]

Hence, the transition of droplet generation between the dripping regime (characterized by monodisperse droplets) and jetting regime (characterized by non-uniform droplet generation) can be controlled by the magnitude of these dimensionless numbers.[3]

SCME was used to approximate droplet diameters and best uniformities for droplets generated when equivalent wall shear was used in xME for scale up at similar Ca_c and We_d.

For the ringed SCME system, the generated membrane wall shear stress has a radial profile with its largest value located on a critical radius (r_c) estimated by:[4,5]

$$r_c = \frac{D}{2}1.23 \left(0.57 + 0.35\frac{D}{T}\right)\left(\frac{b}{T}\right)^{0.036} {n_b}^{0.116} \frac{Re}{1000 + 1.43Re} \text{ m} \tag{S3}$$

and

$$Re = \frac{\rho_c \omega D^2}{2\pi} \tag{S4}$$

where D is the impeller diameter, T the tank diameter, b the blade height, n_b the number of blades, Re the Reynolds number, ω is the angular speed and μ_c is the viscosity of the continuous phase.

For all droplet generation runs with SCME, r_c was found to be within the ringed region. Thus, the average wall shear in the ringed region of the stirred cell (τ_{SCME}) was approximated by:[4]

$$\tau_{SCME} = 1.65\mu_c\omega^{1}/_{\delta}\left[\frac{(r_c^3 - r_1^3)}{3} + \frac{r_c^{1.6}}{1.4}(r_2^{1.4} - r_c^{1.4})\right] \Big/ (r_2^2 - r_1^2) \tag{S5}$$

where r_2 and r_1 are the outer and inner radii of the porous region of the ringed membrane. The boundary layer thickness (δ) was found using the following equation:[4,5]

$$\delta = \sqrt{\frac{\mu_c}{\rho_c \omega}} \tag{S6}$$

As a result of the design of the SCME system which exerts a controlled uniform shear on the membrane surface, SCME was used as a benchmark to ascertain the performance of the cross-flow membrane emulsification (xME) system. Equivalent wall shear (i.e. τ_{SCME}) was used for droplet generation in the xME rig to demonstrate the scalability of the developed formulation and approximate the shear effectiveness of the xME via D_{50} deviations with associated uniformity consequences.

For the xME, the shear was approximated by:[6]

$$\tau_{XME} = \frac{f u_c \rho_c}{2} \tag{S7}$$

where f = wall friction factor = $16/Re$ for $Re < 500;$ or $0.0792Re^{-0.25}$ for $Re < 500$.

Section S3. Particle Stability

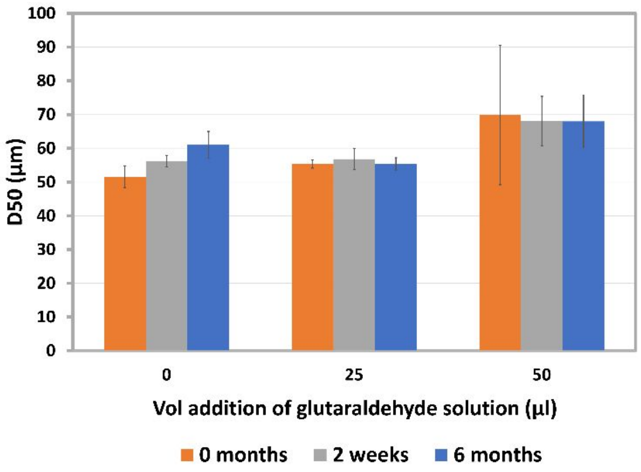

Figure S1. Average particle size (D_{50} by volume) over time of keratin-chitosan microcapsules containing sunflower oil, treated with 0, 25 or 50 µL glutaraldehyde solution per 10 mL sample. Error bars represent standard deviation.

REFERENCES

1. Suárez, M. A.; Gutiérrez, G.; Coca, J.; Pazos, C., Stirred tank membrane emulsification using flat metallic membranes: A dimensional analysis. Chemical Engineering and Processing: Process Intensification 2013, 69, 31-43.

2. Coombs Obrien, J.; Torrente-Murciano, L.; Mattia, D.; Scott, J. L., Continuous Production of Cellulose Microbeads via Membrane Emulsification. ACS Sustainable Chemistry & Engineering 2017, 5 (7), 5931-5939.

3. Bertrandias, A.; Duval, H.; Casalinho, J.; Giorgi, M.-L., Dripping to jetting transition for cross-flowing liquids. Physics of Fluids 2017, 29, 044102.

4. Dragosavac, M. M.; Sovilj, M. N.; Kosvintsev, S. R.; Holdich, R. G.; Vladisavljević, G. T., Controlled production of oil-in-water emulsions containing unrefined pumpkin seed oil using stirred cell membrane emulsification. Journal of Membrane Science 2008, 322 (1), 178-188.

5. Medina-Llamas, M.; Mattia, D., Semi-continuous production of iron oxide nanoparticles via membrane emulsification. Applied Surface Science 2019, 463, 504-512.

6. Holdich, R.; Dragosavac, M.; Williams, B.; Trotter, S., High throughput membrane emulsification using a single-pass annular flow crossflow membrane. AIChE Journal 2020, e16958
